# The Correlation of Adsorption Behavior between Ciprofloxacin Hydrochloride and the Active Sites of Fe-doped MCM-41

**DOI:** 10.3389/fchem.2018.00017

**Published:** 2018-02-07

**Authors:** Ying Wu, Yiming Tang, Laisheng Li, Peihong Liu, Xukai Li, Weirui Chen, Ying Xue

**Affiliations:** ^1^School of Chemistry and Environment, South China Normal University, Guangzhou, China; ^2^Guangdong Provincial Engineering Technology Research Center for Drinking Water Safety, Guangzhou, China; ^3^Guangdong Provincial Key Lab of Functional Materials for Environmental Protection, Guangzhou, China

**Keywords:** ciprofloxacin hydrochloride, Fe-MCM-41, adsorption capacity, pH, complexation, metal cations

## Abstract

**HIGHLIGHTS**

- Fe incorporation significantly accelerated the adsorption of CPX on MCM-41.
- Fe leaching can be ignored when pH was higher than 4.0.
- pH played an important role in CPX adsorption on Fe-MCM-41.
- Co-effect of CPX and metal cations on Fe-MCM-41 was investigated.

Fe-MCM-41s with various molar ratios of silicon to iron (20, 40, 80, and 160) were prepared to investigate adsorption properties of ciprofloxacin hydrochloride (CPX) in aqueous solutions. Fe-MCM-41s were characterized by transmission electron microscope (TEM), X-ray diffraction (XRD), X-ray photoelectron spectroscopy (XPS), nitrogen adsorption/desorption isotherms, and infrared spectroscopy (FT-IR). Effects of silicon-iron ratio, adsorbent dosage, pH, and temperature were conducted to explore the adsorption mechanism of CPX on Fe-MCM-41. The results showed that the introduction of iron facilitated the absorption quantity for CPX from 20.04 to 83.33 mg g^−1^ at 120 min of reaction time, which was mainly attributed to surface complexation. The promotion of hydrophobic effect, electrostatic interactions, and π-π electron donor-acceptor interaction also played coordinate roles in the adsorption process. The experimental kinetic data followed both the pseudo-second-order and intra-particle diffusion models, while the adsorption isotherm data fit well to Freundlich model at high temperature. Thermodynamic study showed that the adsorption was spontaneous. Under the effect of electrostatic interaction, pH of the solution strongly affected CPX adsorption. Five representative metal cations (Ca, Cu, Ni, Pb, and Cd) were chosen to study the effects on CPX adsorption and their complexation. The inhibiting effect of metal cations on CPX adsorption was sequenced in the order of Cu > Ni > Pb > Cd > Ca, which followed the same order as the complexation stability constants between CPX and cations. The Fe-MCM-41 adsorbent possessed excellent reusability for 4 cycles use, suggesting a potential applicability of Fe-MCM-41 to remove CPX in water.

## Introduction

During the last decades, pharmaceuticals and personal care products (PPCPs) among the emerging contaminants have caused more concern of environmental research than the conventional priority pollutants (Arp, 2012; Liu and Wong, 2013; Hayat and Marty, 2014; Montes-Grajales et al., 2017). With abusive use of various antibiotics, large quantity of pharmaceutical wastewater (Cardoso et al., 2014), hospital effluents (Ory et al., 2016; Verlicchi and Zambello, 2016), and excreta-urine (Zheng et al., in press) containing antibiotics have been discharged into environment and regarded as an emerging issue around the world. As a high-use second generation fluoroquinolone, ciprofloxacin hydrochloride (CPX) has the strongest antimicrobial activity and has the highest water concentration in Pearl River of Guangzhou (Bu et al., 2013). Similar to other antibiotics, CPX can transfer in natural environments either as the parent compound or its hydrolysis products, conjugates, oxides when excrete from a target organism (Sarmah et al., 2006) and bring great threats to the ecosystem and human health by inducing proliferation of drug-resistance bacteria (Johnson et al., 2015; Wang et al., 2017b). Therefore, the removal of CPX from water has become a pressing problem. Various methods have been attempted for the removal of CPX from water, such as ultrasound decomposition (Xiao et al., 2013), photocatalytic degradation (Bojer et al., 2017), membrane bioreactor (Hamjinda et al., 2017), ozonation (Gomes et al., 2017), and adsorption on bamboo-based activated carbon (Carabineiro et al., 2012; Peng et al., 2015; Wang et al., 2015), goethite (Gu et al., 2015), graphene oxide (Chen et al., 2015; Fei et al., 2016), Aluminum and Iron hydrous oxides (Gu and Karthikeyan, 2005), and palygorskite-montmorillonite (Berhane et al., 2016). Adsorption technique is widely applied to remove antibiotics from wastewater as a promising method due to its simple theoretical design, ease of operability, relatively low costs and lower amounts of toxic byproducts.

As a member of the M41S family, MCM-41 applied to adsorption area has attracted more awareness because of its hexagonal arrays of uniform channels, high surface area and pore volume and hydrothermal stability (Lee et al., 2007; Jiang et al., 2012). However, hydrophobicity of CPX limits the adsorption capacity by highly hydrophilic MCM-41. Therefore, functional modifications are desired to improve its performance. In the former research, CPX adsorption process can be influenced by metal cations which has been demonstrated that many cations have complexation ability to CPX (Turel et al., 1996). It has been reported that the environmental fate of CPX can be affected by coexisting cations such as Ca and Cu (Pei et al., 2010; Chen et al., 2013). Hence, on the one hand, the introduction of metal ingredient to MCM-41 leads to cation bridging and hydrophobic enhancement, which is beneficial to material for better absorbability. On the other hand, there are few investigations dealing with the different impacts of various free heavy metals on CPX adsorption by comparison.

The main objectives of this paper are to prepare an adsorbent which can enhance the adsorption capacity of CPX by modifying the pure MCM-41 with Fe, to evaluate the effects of various factors including silicon-iron ratio, adsorbent dosage, pH, contact time, temperature, and to study the adsorption mechanisms. Specifically, when CPX coexists with heavy metals in the aqueous phase, the relationship of the formation of Metal cations/CPX complex and the adsorption abilities of CPX is presented to estimate the optimal condition of Fe-MCM-41 adsorbent in practical use.

## Experimental section

### Materials

These reagents were used to synthesize and modify MCM-41: cetyltrimethyl ammonium bromide (CTAB), sodium silicate (NaSiO_3_·9H_2_O) and ferric nitrate (Fe(NO_3_)_3_·9H_2_O) were obtained from Sinopharm Chemical Reagent Co. Ltd. (Shanghai, China). For the adsorption experiments, ciprofloxacin hydrochloride (CPX) was obtained from Macklin Biochemical Co. Ltd. (Shanghai, China). Acetonitrile was chromatography grade and was purchased from Tianjin Kemiou Chemical Reagent Co. Ltd. (Tianjin, China). All the other metals were nitrate salt species and analytical grades. Deionized water was used throughout the study. pH value of the solution was adjusted with 0.1 M hydrochloric acid (HCl) and sodium hydroxide (NaOH).

### Synthesis of adsorbent

MCM-41 was prepared via a hydrothermal treatment using Na_2_SiO_3_·9H_2_O as silicon source and CTAB as the structure-directing group. Briefly, dissolved sodium silicate (0.1 mol) was stirred at 313 K for 15 min. H_2_SO_4_ (2 M) was added dropwise to form a gel. After adding CTAB (7.28 g dissolved in 25 ml water at 338 K) to the gel, the mixed solution was stirring for 30 min, and then aged at 418 K for 48 h. The product gotten was filtered after natural cooling, washed with deionized water and dried. Finally, the sample was calcined for 6 h at 823 K to obtain MCM-41. Fe-MCM-41 was prepared from the same route besides adding Fe(NO_3_)_3_·9H_2_O as iron modification to the solution before pH adjustment. The samples were designated as Fe-MCM-41(x) (x = 20, 40, 80, and 160), where x was denoted as different molar ratios of Si to Fe for as-synthesized materials.

### Analytical procedures

The low-angle XRD measurements (D8 ADVANCE, BRUKER, German) were carried out in the 2θ ranges of 1.0–8.0°. The N_2_ adsorption/desorption isotherms (ASAP2020, Micromeritics, USA) was used to calculate the surface area. The pH_pzc_ value was measured by potentiometric titration. The leaching iron content of the solution was determined by atomic absorption Spectrophotometer (AA-7020, EWAI, China). The FT-IR absorption spectra (6700, Nicolet, USA) was obtained over the range 4,000–400 cm^−1^.

The concentration of CPX was quantified using HPLC (LC10A, Shimadzu) consisting of a UV detector (SPD-10AV) at 278 nm and a Diamonsil C18 column (250 × 4.6 mm, 5 μm, Dikmate technologies). The mobile phase used for detection of CPX was acetonitrile/0.01 M potassium dihydrogen phosphate (23:77, v/v) and the flow rate was 1.0 mL min^−1^.

### Batch adsorption experiments

For adsorption experiments, 0.04 g of adsorbent was mixed with 200 mL of CPX solution (20 mg L^−1^, initial pH = 5.40) in flasks and the mixture stirred at 303 K up to beyond the equilibrium time. The rotating speed was set to 175 rpm. Additional experiments were also performed to study the effects of equilibrium time, dosage, pH, temperature and metal cations on the adsorption properties. To study the impact of various metal cations on the adsorption behavior of CPX, predetermined amounts of Ca(II), Cu(II), Ni(II), Pb(II), Cd(II) were introduced to obtain 0.01 M ionic strength solutions at 303 K, respectively. Controls without Fe-MCM-41 were considered to explain losses from adsorption to flasks. All experiments were run in triplicate under the same conditions.

A series of adsorbent doses from 30 to 60 mg were selected to discuss the effect of dosage. The pH effects experiments were conducted by adjusting the pH between 3 and 11. To study the impact of various metal cations on the adsorption behavior of CPX, predetermined amounts of Ca(II), Cu(II), Ni(II), Pb(II), Cd(II) were added to obtain 0.01 M ionic strength solutions at 303 K, respectively. The isotherms were obtained by batch experiments performed at 293 ± 0.5, 303 ± 0.5, 313 ± 0.5 K, respectively. The initial concentrations of CPX (varying from 20 to 80 mg L^−1^) were chosen based on preliminary experiments, which controlled the adsorbed amount of CPX between 30 and 90% of the initial amounts. A study of reusability was carried out by adsorption/desorption for four times.

## Results and discussion

### Characterization of adsorbents used in this work

**Figure 1:**
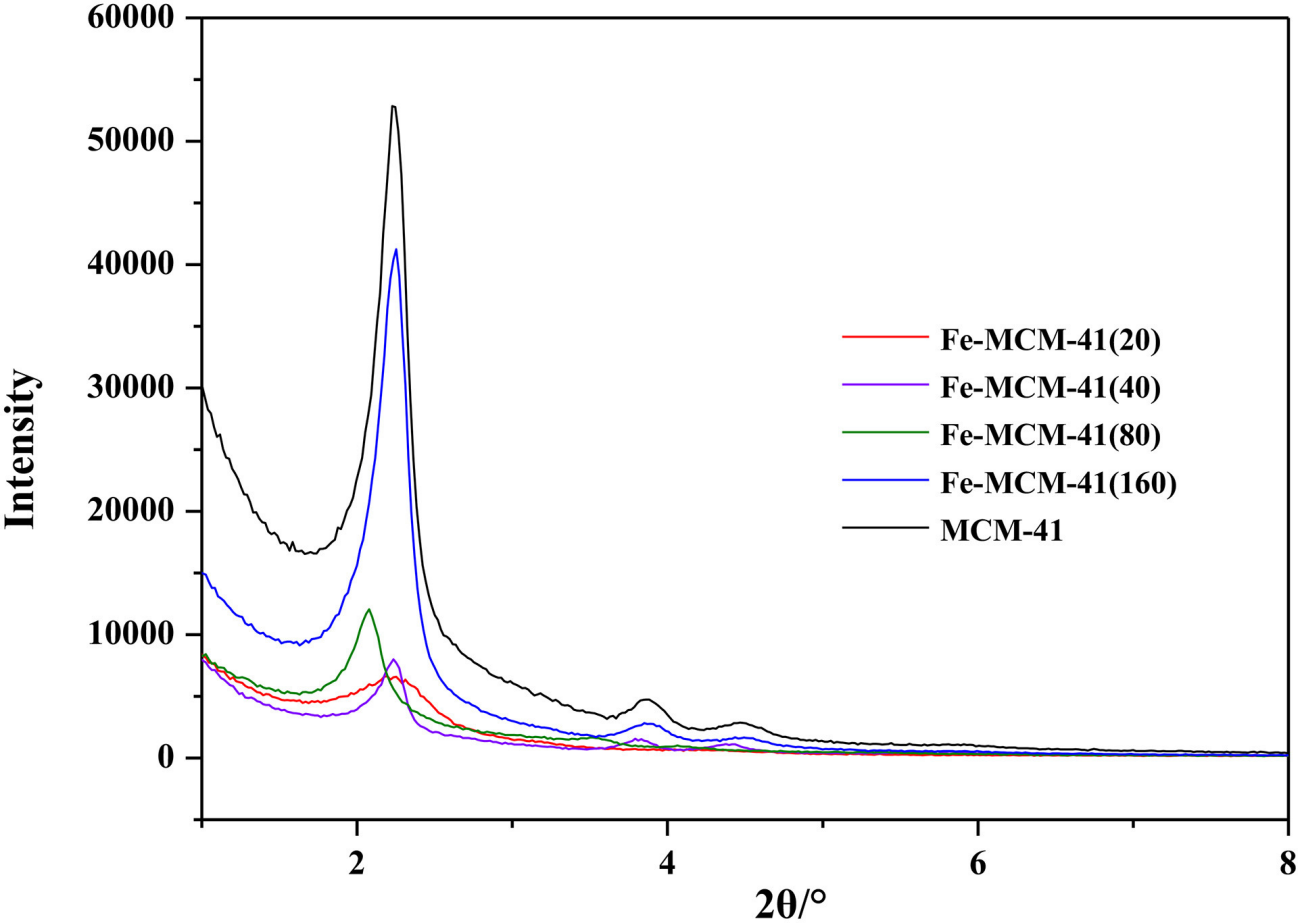
The XRD pattern of Fe-MCM-41(x) (x = 20, 40, 80, and 160, x was denoted as molar ratio of Si to Fe).

Figure 1 showed the low angle XRD patterns of all absorbents at different molar ratios of Si to Fe and pure sample. All absorbents had three well-resolved peaks indexed to (100), (110), and (200) diffraction planes, indicating a highly ordered hexagonal mesostructure (Li et al., 2012). When the silicon-iron ratio decreased, iron content became higher, the intensity of the peaks diminished and the peak width increased. This meant the introduction of excess iron could reduce ordering of the structure. The result of the segment was consistent with the TEM exhibited in Figure S1.

**Figure 2:**
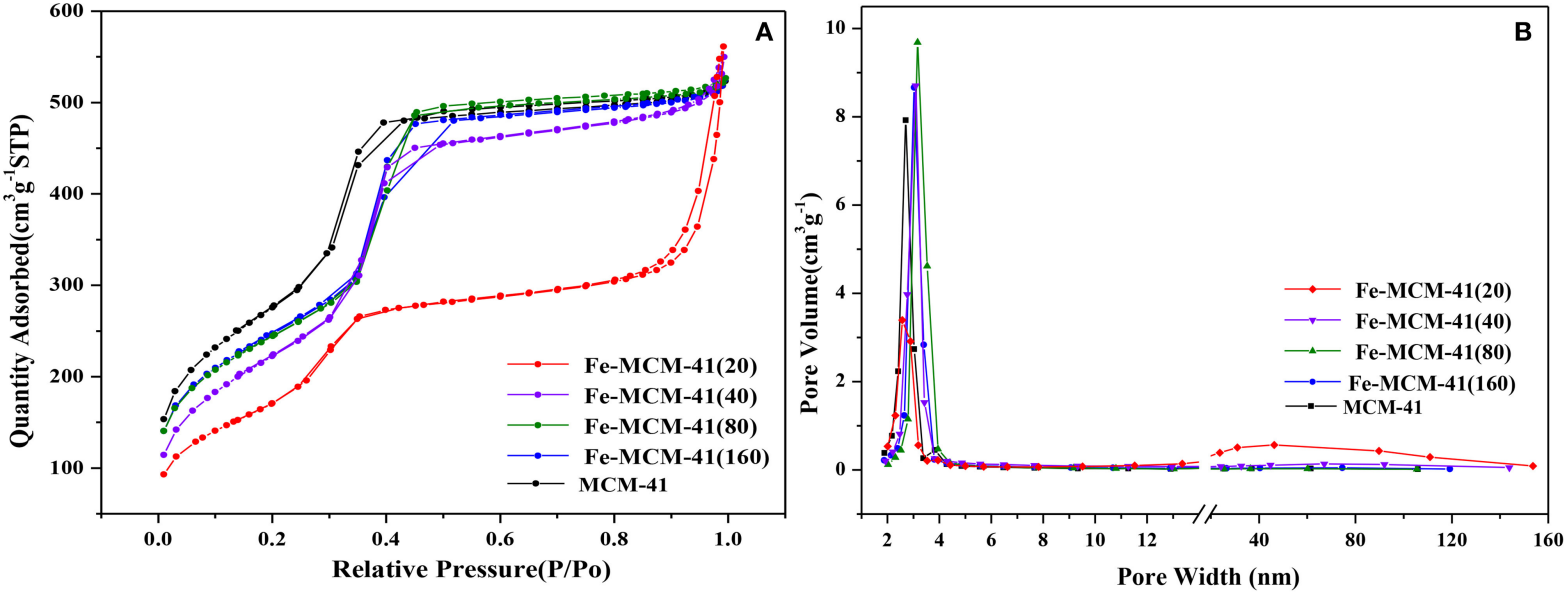
**(A)** The nitrogen adsorption/desorption isotherms, and **(B)** pore size distributions of Fe-MCM-41(x) (x = 20, 40, 80, and 160, x was denoted as molar ratio of Si to Fe).

**Figure 3:**
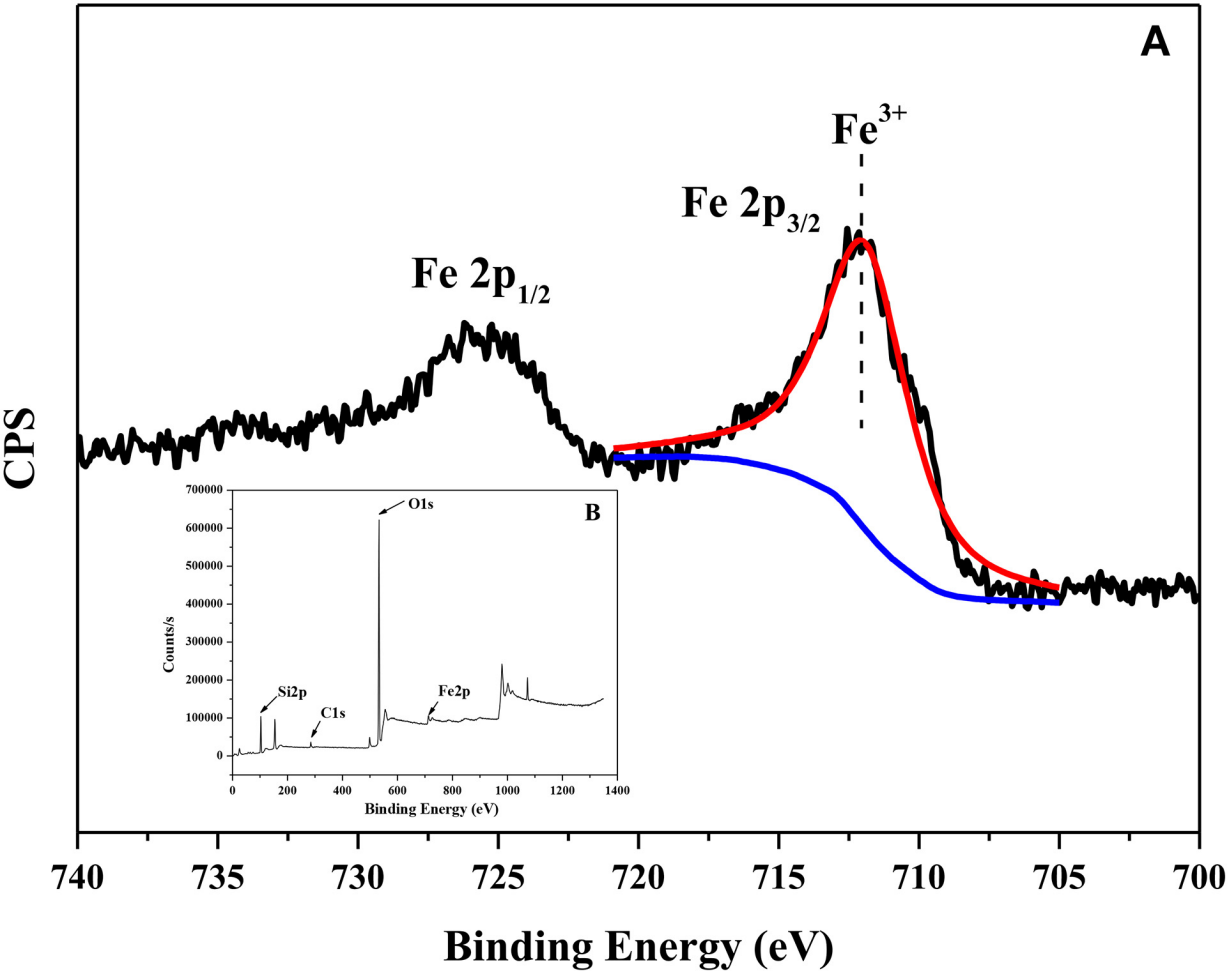
XPS spectrum of Fe-MCM-41.

All isotherms in Figure 2 belonged to type IV isotherm according to IUPAC classification. Compared with the other four absorbents, the BET curve and the BJH pore size distribution curve of Fe-MCM-41(20) exhibited obviously different. The position closed to p/p_0_ = 0.9 showed a distinct region of steep increase, which was corresponding to the capillary condensation in the pristine silica gel pores (Kolesnikov et al., 2017). This was similar to the above discussion of XRD that the pores size increased after iron-doping during aging process gradually. Except for tiny amounts of macroporous structures, the vast majority of pore widths were below 5 nm. It could be seen from the structural properties of all absorbents in Table S1. The pore diameters increased from 3.15 to 4.33 nm and the BET surface area decreased from 1002.1 to 625.6 m^2^ g^−1^. Note that by replacing Si^4+^ (0.40 Å) with Fe^3+^ (0.63 Å) in the structure of material, the extension of radius enlarged the pore sizes and decreased surface area. Moreover, XPS was provided to clarify the chemical phase of Fe species in the synthesized Fe-MCM-41. XPS spectrum in Figure 3 gave a description that the peaks at 712 and 725 eV for the binding energies of Fe2p_3/2_ and Fe2p_1/2_, reflecting iron species existed as Fe^3+^ in Fe-MCM-41 (Nie et al., 2015).

### Effect of silicon-iron ratio

**Figure 4:**
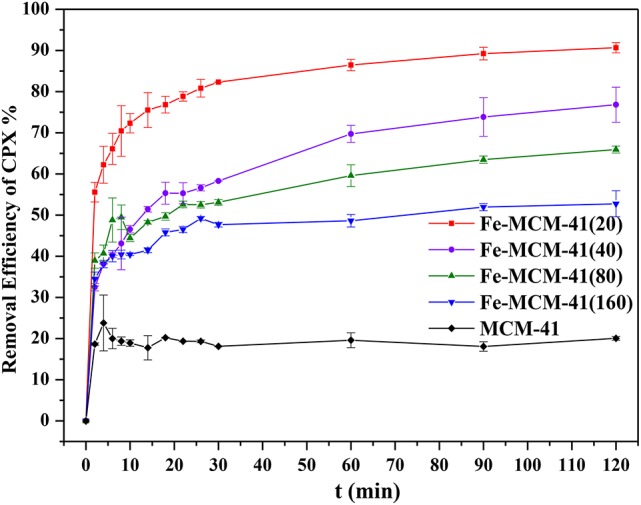
Adsorption of CPX on Fe-MCM-41(x) (x = 20, 40, 80, and 160, x was denoted as molar ratio of Si to Fe; volume of CPX solution: 200 mL; rotating speed: 175 rpm/min; initial pH value: 5.40; equilibrium time: 120 min).

**Figure 5:**
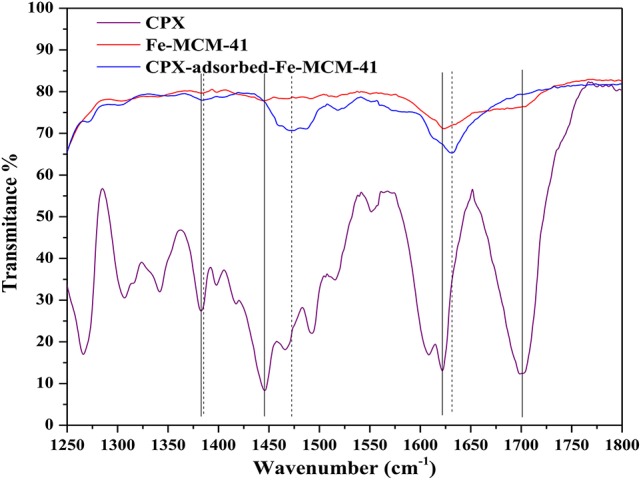
FT-IR spectra of CPX, Fe-MCM-41 and CPX-adsorbed Fe-MCM-41. The characteristic band positions of pure CPX were marked by black lines. The distinctive band positions of CPX as a result of adsorption on Fe-MCM-41 were denoted by dashed lines.

In Figure 4, the removal efficiency for CPX decreased with the increasing surface area of Fe-MCM-41. Hydrophobic compound was difficult to be adsorbed on the surface of mesoporous MCM-41 silica which contained large amounts of Si–OH (Hu et al., 2014), and iron doping strengthened the surface hydrophobicity. More importantly, CPX with the numerous reactive functional groups like carboxyl, keto, and piperazine could form complexes with majority of metal cations (Turel, 2002; Sun et al., 2014). As seen in Figure 5, the interactions between CPX and Fe-MCM-41 were investigated by comparing the FT-IR spectra of CPX, fresh and used Fe-MCM-41 in the range of 1250–1800 cm^−1^, which included the main characteristic peaks (Full scale FT-IR curve could be found in Figure S2 in Supplementary Information). The band positions and their corresponding band assignments were listed in Table 1 (Gu and Karthikeyan, 2005; Trivedi and Vasudevan, 2007; Wang et al., 2011; Peng et al., 2012). The position of C=O stretching vibration of pyridine-keto upshifted slightly to a higher frequency of 1631.4 cm^−1^ after adsorption and the 9.4 cm^−1^ shift indicated reinforcement of the C=O bond. That was ascribed to the release of intramolecular hydrogen bond between the ortho substituted carboxylate group and Fe-MCM-41 which was an indirect evidence for ferric-ciprofloxacin interactions. Meanwhile, the intensity of this peak was much weaker, suggesting that the fraction of pyridine-keto group interacted with Fe-MCM-41. The most significant feature of CPX adsorbed on Fe-MCM-41 was the complete disappearance of the peak at 1698.7 cm^−1^, which indicated that all the carboxyl groups participated in the binding reaction with Fe-MCM-41. The band shifting from 1382.8 to 1384.2 cm^−1^ demonstrated that electrostatic interactions were occurred between the negative sites on the absorbent surface and the protonated amine group. The shift exhibited by the band between 1445.8 to 1474.6 cm^−1^ meant the C–N bond of amine group also supported this assumption. Overall, it was almost certain that iron doping added the sites of adsorption (see Graphical Abstract). The complex was present through the cation bridging of Fe(III) with the adjacent carbonyl oxygen and the oxygen from the deprotonated carboxylate group. The other absent peaks after adsorption might show signs of other adsorption affinity, for instance, hydrophobic effect and π-π interaction (discussed later). Fe-MCM-41 (20) was chosen as the adsorbent for the subsequent research.

**Table 1:** FT-IR band positions (cm^−1^) and suggested assignments for CPX and CPX-adsorbed samples.

**CPX**	**CPX-adsorbed samples**	**Band assignment**
1621.85	1631.24	υ (pyridine-keto C = O)
1698.69	–	υ (carboxylic acid C = O)
1445.81	1474.64	υ (Stretching of C–N)
1382.75	1384.16	Protonation of amine group in the piperazine moiety

*υ, stretching; δ, bending*.

**Graphical Abstract:**
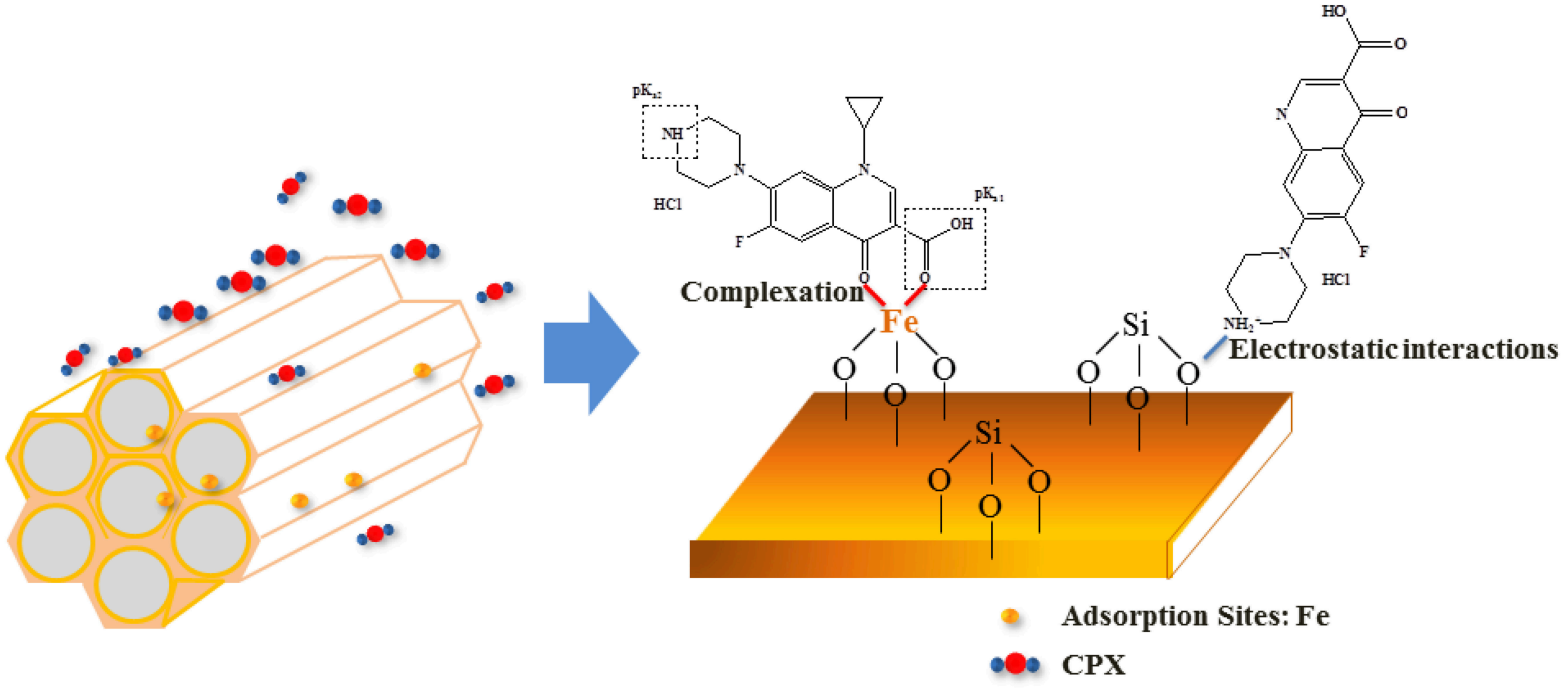
Schematic diagram of adsorption process of CPX by Fe-doped MCM-41 catalyst.

### Influence of adsorbent dose and kinetics of adsorption

**Figure 6:**
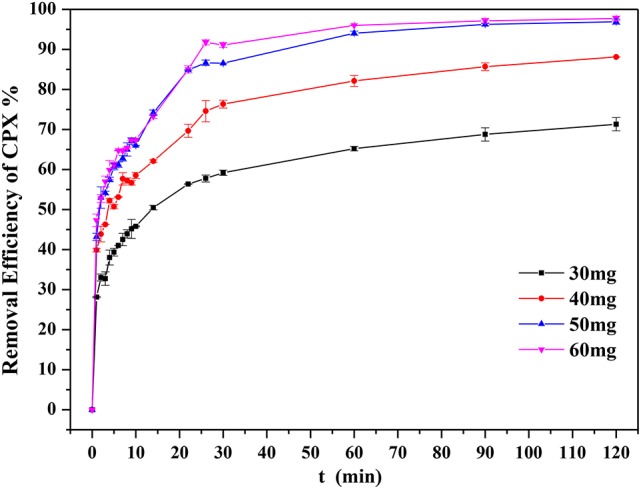
Influence of adsorbent dosage (volume of CPX solution: 200 mL; initial concentration = 20 mg L^−1^; rotating speed: 175 rpm/min; temperature: 303 K; initial pH-value: 5.40).

Figure 6 illustrated the influence of adsorbent dose on the uptake of CPX. It was displayed that the removal efficiency increased with adsorbent dose. This behavior could be explained by adsorption mainly occurring at active sites when the adsorbent dose was large. The high removal efficiency for CPX reflected the satisfactory textural and structural properties of the adsorbent. Therefore, 40 mg adsorbent dose was chosen for the following study.

The kinetics could be employed to evaluate the performance of the adsorbent and give the underlying adsorption mechanisms. Three available kinetic models were examined as follows:

Pseudo-first-order kinetic equation:

(1) $$\frac{1}{q_{t}}=\frac{k_{1}}{q_{e}}\times \frac{1}{t}+\frac{1}{q_{e}}$$

Pseudo-second-order kinetic equation:

(2) $$\frac{t}{q_{t}}=\frac{t}{q_{e}}+\;\frac{1}{k_{2}\times {q_{e}}^{2}}$$

Where *q*_*e*_ and *q*_*t*_ were the adsorption amounts at equilibrium and time t, respectively. *k*_1_ and *k*_2_ were defined as reaction rate constants.

Intra-particle diffusion model:

(3) qt=ki×t12+ci

Where *k*_*i*_ was defined as rate constant at stage *i, k*_*i*_ could be obtained by plotting *q*_*t*_ vs. *t*^1/2^.

**Figure 7:**
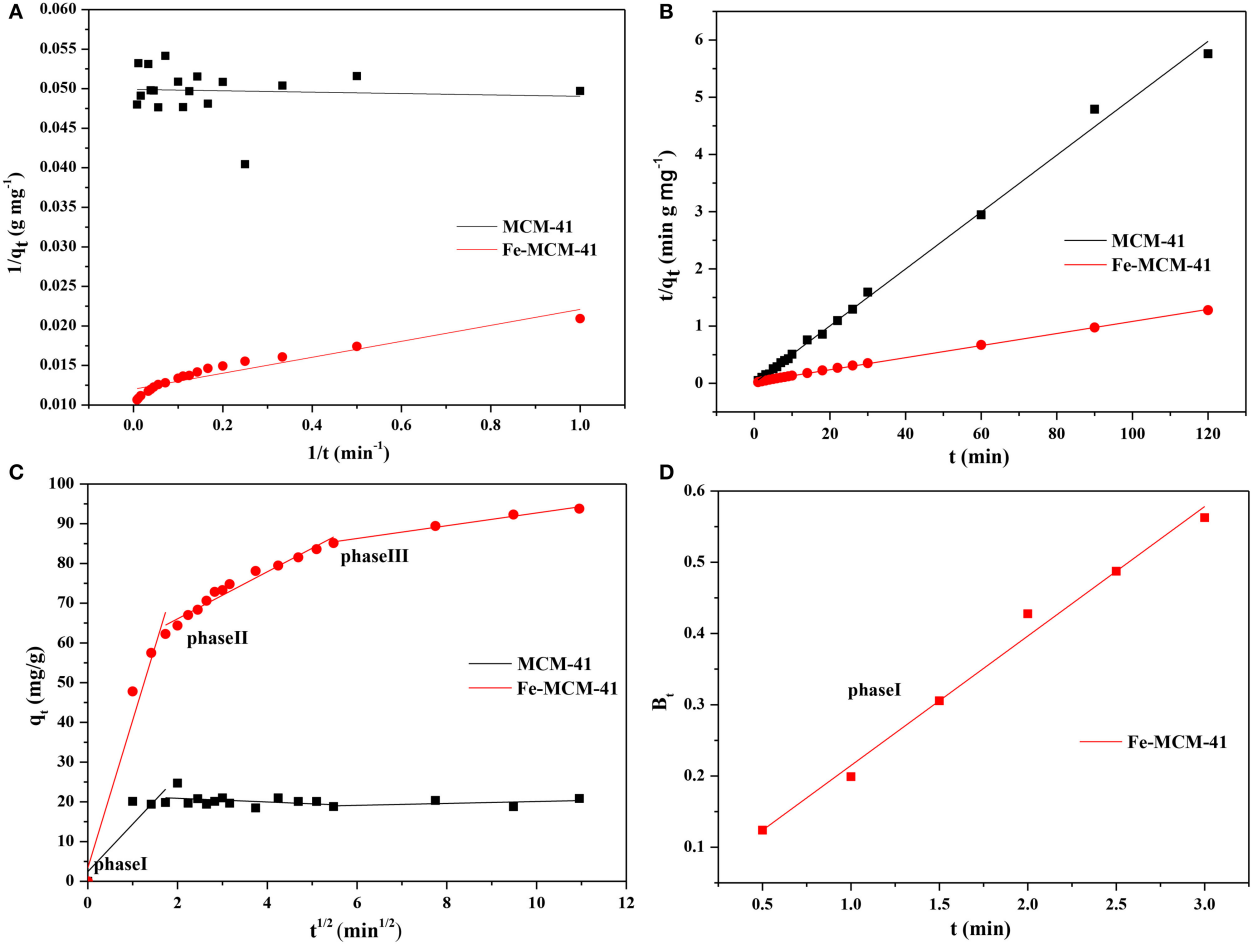
The adsorption Kinetics of CPX by MCM-41 and Fe-MCM-41 **(A)** pseudo-first-order model, **(B)** pseudo-second-order model, **(C)** intra-particle diffusion model and **(D)** Boyd plots for CPX adsorption at different temperatures.

The avidity of three kinetic models was analyzed by fitting straight lines in Figures 7A–C. Table 2 listed the kinetics parameters for three reaction orders. It was obvious that the experimental data of CPX adsorbed by Fe-MCM-41 were more accurately simulated by pseudo-second-order model with higher correlation coefficients (*R*^2^) value. Also, based on the second-order model, the calculated *q*_*e*_ (q_e,cal_) was a good agreement with the experimental *q*_*e*_ (q_e,exp_) values, besides *q*_*e*_ (q_e,exp_) increased from 20.12 to 95.24 mg g^−1^ with Fe content. Compared the various adsorption capacities of CPX with those reported adsorbents such as Graphene oxide/calcium alginate (GO/CA) (Wu et al., 2013), Carbon nanofibers (Li et al., 2015), Goethite (MacKay and Seremet, 2008) and Illite (Wang et al., 2011), H_2_Ti_2_O_5_·H_2_O (Wu et al., 2014), and Fe_3_O_4_/C (Shi et al., 2013) as showed in Table 3, Fe-MCM-41 showed better adsorption property which required a shorter time to reach adsorption equilibrium and possessed a higher adsorption capacity.

**Table 2:** Kinetic parameters for the adsorption of CPX on MCM-41 and Fe-MCM-41.

**Kinetic model**	**Parameters**	**Values**	
		**MCM-41**	**Fe-MCM-41**
Pseudo-first-order	q_e,cal_ (mg g^−1^)	20.0361	83.3333
	*k_1_* (min^−1^)	−0.0175	0.8417
	*R*^2^	−0.0573	0.8950
Pseudo-second-order	*k_2_* (g mg^−1^ min^−1^)	0.2775	0.0043
	*R*^2^	0.9996	0.9993
	q_e,exp_ (mg g^−1^)	20.8354	93.7965
	q_e,cal_ (mg g^−1^)	20.1207	95.2381
Intra-particle diffusion	K_I_	11.9766	37.1644
	C_I_	2.4231	3.3595
	*R*^2^	0.7465	0.9408
	K_II_	−0.4644	5.9203
	C_II_	21.8282	54.2310
	*R*^2^	0.0572	0.9603
	K_III_	0.2410	1.6064
	C_III_	17.6776	76.6507
	*R*^2^	−0.0625	0.9796

**Table 3:** Adsorption capacities of CPX on various adsorbents.

**Adsorbents**	**Adsorption amounts (mg g^−1^)**	**Rate constant of Pseudo second-order kinetic equation (g mg^−1^ min^−1^)**	**References**
Illite	36.781	0.00449	Wang et al., 2011
Graphene oxide/calcium alginate (GO/CA)	12.1124	0.1604	Wu et al., 2013
Carbon nanofibers	639.602	0.00093	Li et al., 2015
Goethite	3.678	–	MacKay and Seremet, 2008
H_2_Ti_2_O_5_·H_2_O	14.81	–	Wu et al., 2014
Fe_3_O_4_/C	38.61	–	Shi et al., 2013
Fe-MCM-41	93.7965	0.0043	This work

The models discussed above were not able to describe the phenomena of intraparticle or pore diffusion, which were often the rate-limiting steps in an adsorption process. With the intra-particle diffusion model, Figure 7C exhibited that the adsorption plots of adsorbents were not linear and could be divided into three linear regions, denoting that the multi stages of the intraparticle adsorption (Ghaedi et al., 2012). The first-stage portion was sharper and did not pass through zero point, which was due to the diffusion of adsorbents through the bulk solution to the external surface. It demonstrated that particle diffusion was not only the sole rate-limiting step, but also controlled by boundary layer in the initial phase of the adsorption. These results could be validated by further analyzing data with Boyd's mode which was expressed as follows:

(4) $$F=1-\frac{6}{\pi^{2}}\exp(-Bt)$$

(5) $$F=\frac{q_{t}}{q_{e}}$$

(6) Bt=−0.4977−ln (1−F)

Where *q*_*t*_ and *q*_*e*_ were the adsorption amounts at any time t and equilibrium, *F* given by Equation (5) was the fraction at time *t, B*_*t*_ denoted a mathematical function of *F*.

The linearity of the plot was applied to differentiate the controlled rates of adsorption between external transport and intra-particle diffusion. The plot in Figure 7D were linear and did not move toward the zero point, demonstrating that external transfer governed adsorption process at the start and then the intra-particle diffusion.

The second-stage portion became slower than first stage due to the weak adsorption between CPX and the surface atoms of the solid. The third-stage portion tended to ease up and reached final equilibrium. It could be attributed to the smaller pores, lower concentration of CPX and enhanced electrostatic repulsion (between CPX and the adsorbents surface). What's more, comparisons of the values of k_i_ also proved that the adsorption rate became slower during the adsorption process. It concluded that the intraparticle diffusion was present while some other mechanisms might be involved.

### Effect of the temperature

**Figure 8:**
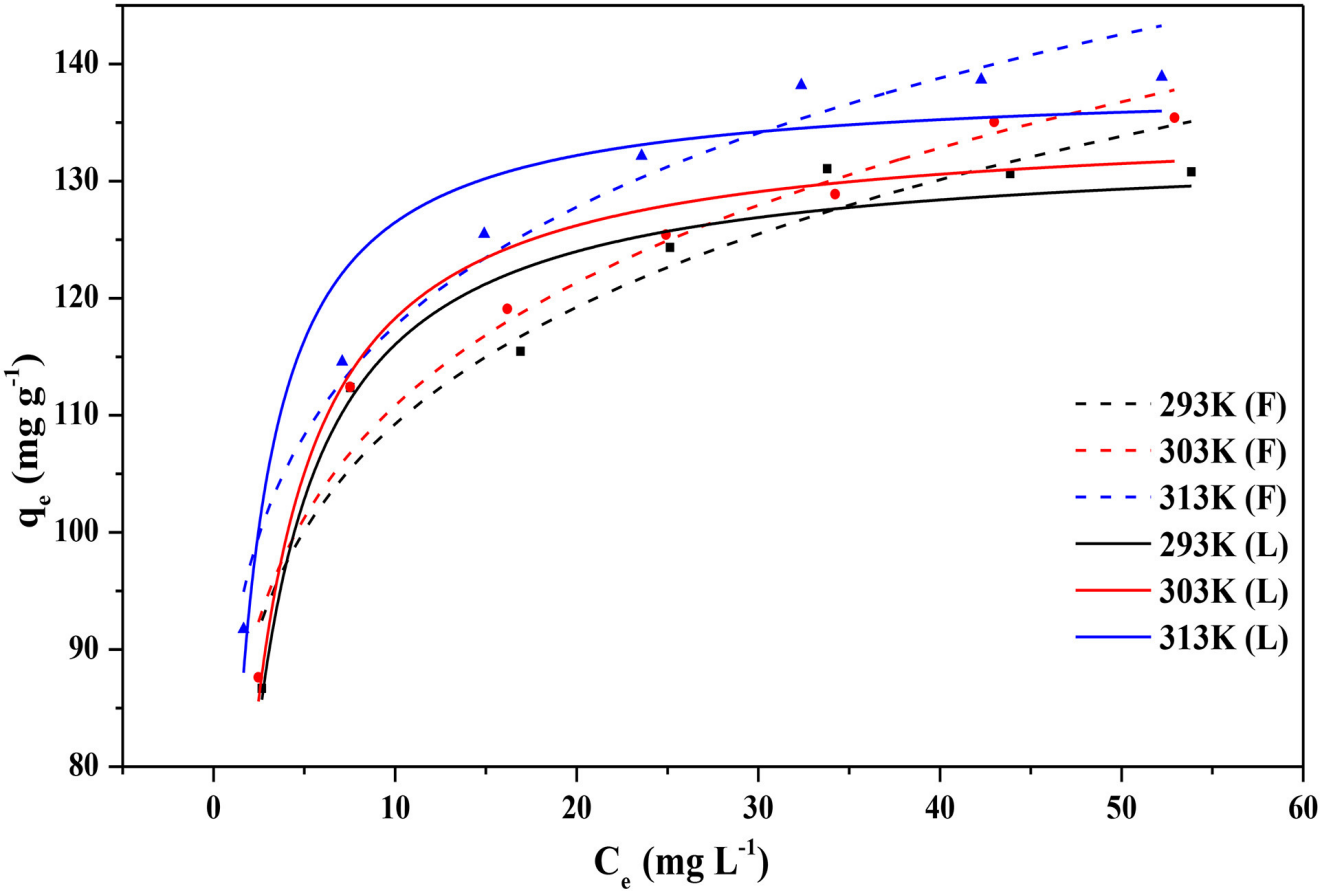
The adsorption isotherm of CPX on Fe-MCM-41(20), solid lines represent Langmuir model fits to the data, dotted lines represent Freundlich model fits to the data (volume of CPX solution: 200 mL; rotating speed: 175 rpm/min; initial pH-value: 5.40; equilibrium time: 120 min).

The adsorption isotherm in Figure 8 described the equilibrium relationships between the liquid-phase CPX concentration and the amount of CPX on the Fe-MCM-41. The adsorption capacities of the CPX increased with temperature, indicating that adsorption process on Fe-MCM-41 was favored at higher temperatures. The phenomenon could be ascribed to promote mobility of the CPX molecules in solution and increased new active sites on the Fe-MCM-41 for adsorption (Ghaedi et al., 2012). The results also suggested that it was an endothermic process in nature.

For further examining the impact of temperature on adsorption process, thermodynamic parameters was determined at three temperatures (Ao et al., 2009). As listed in Table 4, the adsorption parameters were fitted by the most frequently utilized adsorption isotherms models named Langmuir and Freundlich models at 293, 303, and 313 K. It was apparent that the Langmuir isotherm better represented the experimental data with higher value of the determination coefficient (*R*^2^ = 0.9459) at 293 K while Freundlich model yielded the best fit at temperatures 303 K (*R*^2^ = 0.9544) and 313 K (*R*^2^ = 0.9675). This implied monolayer adsorption with a value of 133.33 mg g^−1^ obtained for q_max_ and evenly distribution of the adsorption sites at low temperature. However, as temperature rose, the Freundlich equation became even more accurate to assess the adsorption process. It was possible to deduce that the thermal treatments activated more active sites for adsorption as described above. Molecules of CPX retained two monolayer adsorptions when the pore diameter of Fe-MCM-41 was greater than molecule diameter of CPX, and despite reversible adsorption occurred meanwhile (Song et al., 2013; Wang et al., 2017a). This non-linear adsorption also demonstrated that the adsorption process was the result of interaction of various forces. The increased values of K_F_ with temperature demonstrated the endothermic adsorption process. The *n*-values were more than 1, revealing a beneficial adsorption system and heterogeneity of Fe-MCM-41 adsorption sites (Wu et al., 2017).

**Table 4:** Adsorption isotherms constants and correlation coefficients.

**T (K)**	**Langmuir** $\frac{1}{q_{e}}=\frac{1}{q_{m}\times k_{L}\times Ce}+\frac{1}{q_{m}}$			**Freundlich** qe=KF×Ce1n		
	**K_L_ (L mg^−1^)**	**q_m_ (mg g^−1^)**	***R*^2^**	**K_F_ (mg g^−1^)/(mg L^−1^)^1/n^)**	***n***	***R*^2^**
293	0.701	133.333	0.9459	71.4140	7.513	0.9080
303	0.74	135.135	0.9497	70.8384	7.337	0.9544
313	1.177	136.986	0.9167	78.3907	8.071	0.9675

As listed in Table 5, the thermodynamic parameters, such as enthalpy change (Δ*H*), entropy change (Δ*S*), and the Gibb's free energy (Δ*G*), were evaluated to obtain insights into the changes of adsorption processes using the Van't Hoff equation (Jiang et al., 2013). The value of *K*_*d*_, an equilibrium constant, was obtained by dividing *q*_*e*_ and *C*_*e*_. The thermodynamic parameters of adsorption could be determined by using Equations (7) and (8):

(7) $$\Delta G=-R\times T\times \text{ln }K_{d}$$

(8) $$\text{ln }K_{d}=\frac{\Delta S}{R}-\frac{\Delta H}{R\times T}$$

The negative Δ*G*-values were obtained in the range of 293–313 K, revealing the feasible and spontaneous of adsorption process. The positive Δ*H*-value demonstrated an endothermic process while the positive value of Δ*S* indicated that the organization of the CPX molecular at the solid- liquid interface became more random than low temperature.

**Table 5:** Thermodynamic parameters for the adsorption under different temperature.

**T (K)**	**ΔG (KJ mol^−1^)**	**ΔH (KJ mol^−1^)**	**ΔS (KJ mol^−1^ K^−1^)**
293	−7.5041	9.926	59.2
303	−7.8718		
313	−8.6888		

### Effect of pH on the adsorption of CPX

**Figure 9:**
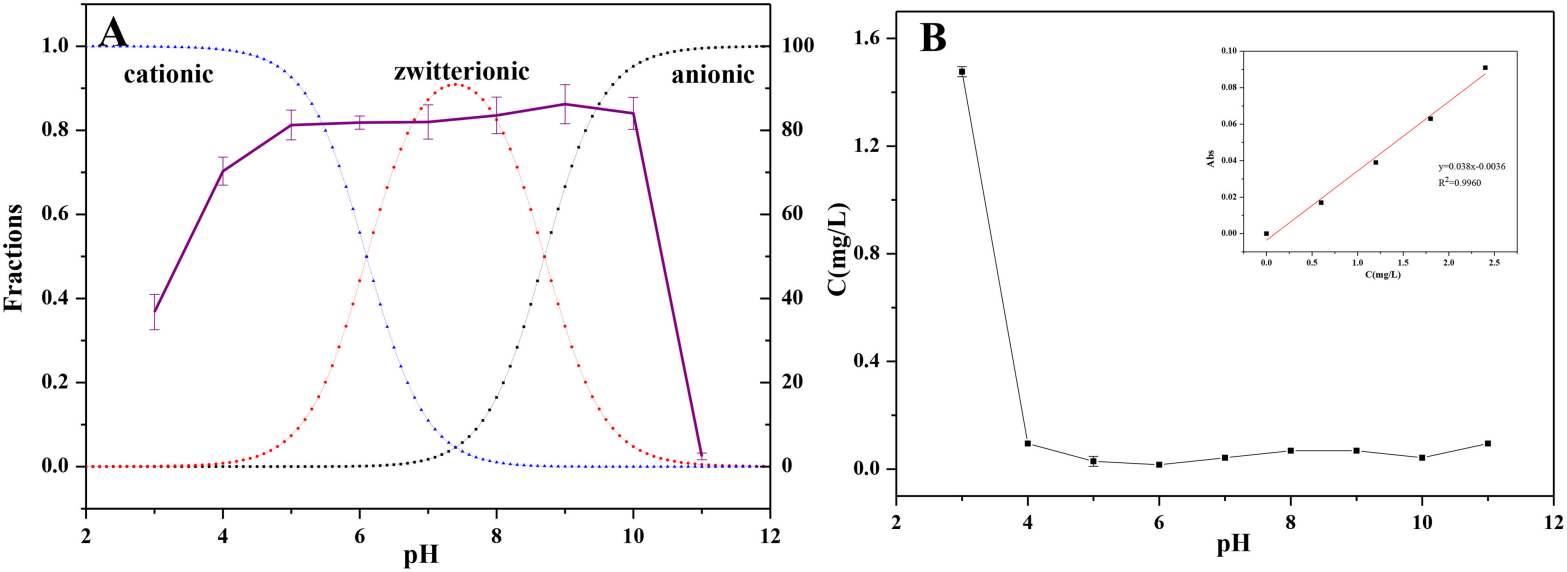
**(A)** The relation of removal efficiency of CPX to the pH values of Fe-MCM-41 at 298 K (The dotted lines represented the fraction of cationic, zwitterionic, and anionic forms of CPX), **(B)** the leaching of Fe content at different pH-value.

The pH_pzc_ of Fe-MCM-41(20) was estimated to be around 5.4, the surface charge of Fe-MCM-41(20) remained negative over the range of pH (5.4 < pH < 11.0) in this study. Therefore, the speciation of CPX molecules were regarded as the mainly factor to this pH range (5.4 < pH < 11.0) influence on the adsorption level. Figure 9 indicated the pH dependency of CPX adsorption on Fe-MCM-41(20). As seen there, a bell-shaped adsorption envelop was noticed. Initially, adsorption increased with pH from 5 to 10 and reached to a maximum value. At pH values >10, adsorption decreased sharply with increasing pH. The acid dissociation constants pK_a1_ and pK_a2_ values of CPX were 6.1 and 8.7, respectively. Most of CPX molecular were in cationic form with protonated amine group in the piperazine moiety when pH was less than 6.1. Cationic CPX generated electrostatic attraction with negatively charged Fe-MCM-41(20) on the surface, accordingly promoting the adsorption. As displayed in Figure 9B, a great amount of Fe leached and the structure of adsorbent collapsed at strong acidic environment (pH < 4), which greatly reduced the adsorption ability. When 6.1 < pH < 8.7, CPX removal efficiency increased with its zwitterion form. The zwitterionic CPX was least soluble at pH 7.5 in water for its neutral charge (Roca Jalil et al., 2015). In other word, it could be assumed that hydrophobic effect had played an important role in the adsorption process. When pH value was above 8.7, the anionic form could induce the electrostatic repulsion between CPX and Fe-MCM-41. When 8.7 < pH < 10, another force might exist to balance the hydrophobic effect and electrostatic repulsion which suggested as π-electron-donor–acceptor (EDA) interaction between CPX and Fe-MCM-41(20). The aromatic ring could serve as π-electron acceptors due to the strong electron-withdrawing ability of fluorine group on the benzene ring of CPX. In this, the hydroxyl groups on adsorbent surface acting as electron-donors interacted strongly with the π-acceptor compound. When pH > 10, a drastic decrease of CPX adsorption amount was due to the strong electrostatic repulsion effect. Therefore, strong chemical interactions were of vital importance for CPX adsorption on Fe-MCM-41(20).

### The effect of metal cations

**Figure 10:**
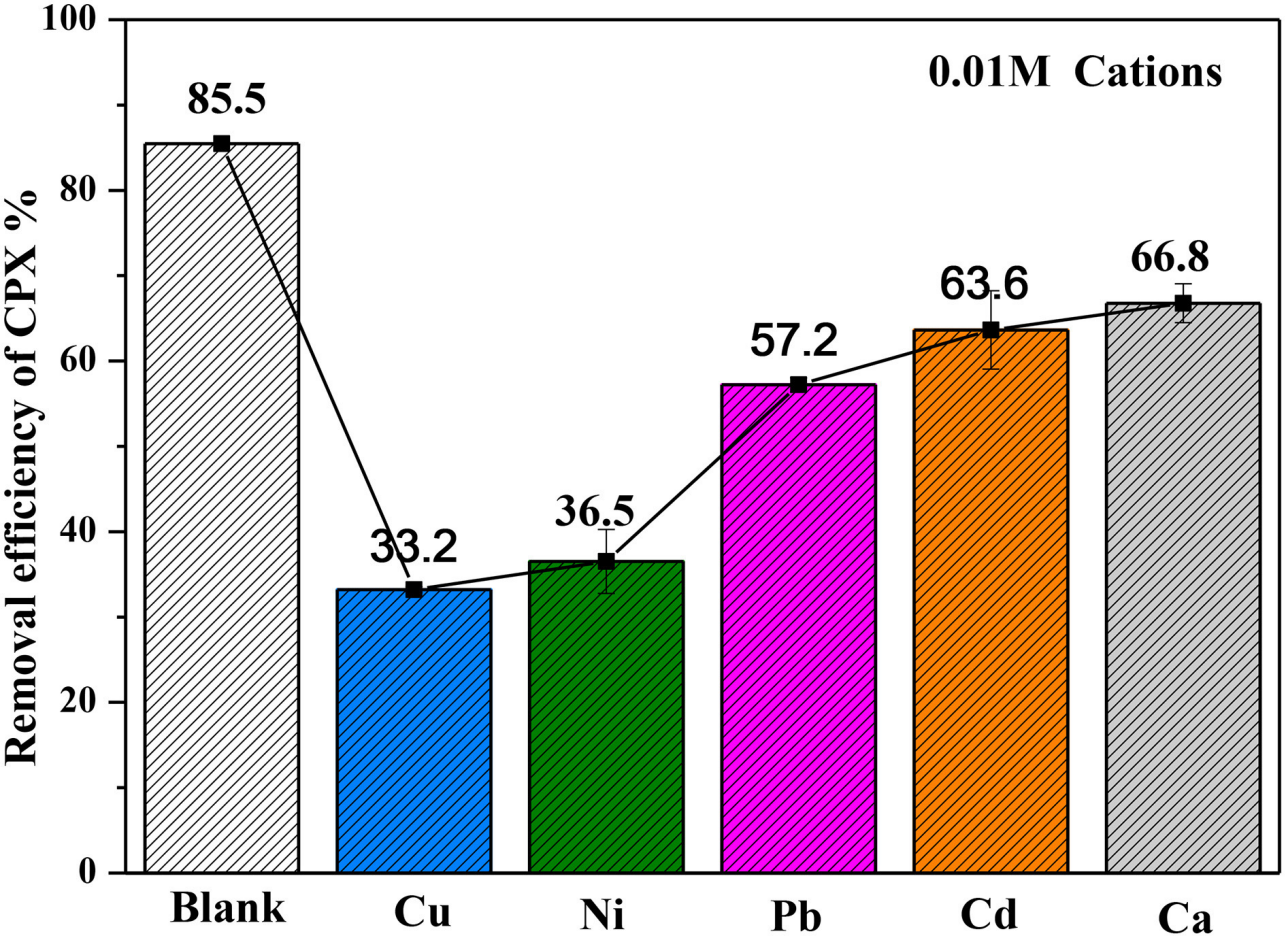
Influence of metal cations on removal efficiency of CPX (volume of CPX solution: 200 mL; rotating speed: 175 rpm/min; initial pH-value: 5.40; equilibrium time: 120 min).

Metal cations with relatively high concentration could impact CPX adsorption. The presence of Cu(II) facilitated the adsorption of CPX on montmorillonite at pH > 6.0 (Pei et al., 2010). Similar conclusion was also given on activated carbon at pH 3.4–6.5 (Sun et al., 2014). As depicted in Figure 10, all metal cations suggested a significantly decline to the adsorption property of CPX, whether it be Ca represented alkaline earth element or those metals which were common divalent metals (Cu, Ni, Pb, and Cd) in water. The influence of five metal cations on removal efficiency followed a decreasing order of Cu (52.3%) > Ni (49.0%) > Pb (28.3%) >Cd (21.9%) >Ca (18.7%). This phenomenon could be assumed that metal cations screened negative charged sites of adsorbent surface. Metal cations had a relatively strong affinity to CPX. In order to make a thorough exploration about the complexing ability of five metal ions, the complexation stability constants were listed in Table 6 (combined with the prior results). Some conclusions can be drawn from the values. On the basis of the liberation degrees of CPX, which complexed with metal cations mainly in three forms: [M(AH)]^2+^, [M(AH)_2_]^2+^, [M(AH)A]^+^. Because of the solution under neutral conditions, [M(AH)]^2+^ was the main form. Compared to other divalent metals, the complexation ability of CPX with Cu was much stronger, and the result agreed with the heavy metals which exhibited different removal efficiency.

**Table 6:** Complexation constants of CPX with metals found in the literature.

**Equilibria**	**Ca**	**Cu**	**Ni**	**Cd**	**Pb**	**References**
M^2+^+HA^±^ = [M(AH)]^2+^	2.75	6.19	4.39			Turel et al., 1996
[M(AH)]^2+^+HA^±^ = [M(AH)_2_]^2+^		5.26	4.3			
[M(AH)_2_]^2+^ = [M(AH)A]^+^+H^+^		−6.6	−7.35			
M^2+^+HA^±^ = [M(AH)]^2+^	2.75	6.14		3.14	3.86	Tan et al., 2014
[M(AH)]^2+^+HA^±^ = [M(AH)_2_]^2+^		10.49		5.01	7.51	
[M(AH)_2_]^2+^ = [M(AH)A]^+^+H^+^		3.32		−1.12	0.38	

### Regeneration of the adsorbent

**Figure 11:**
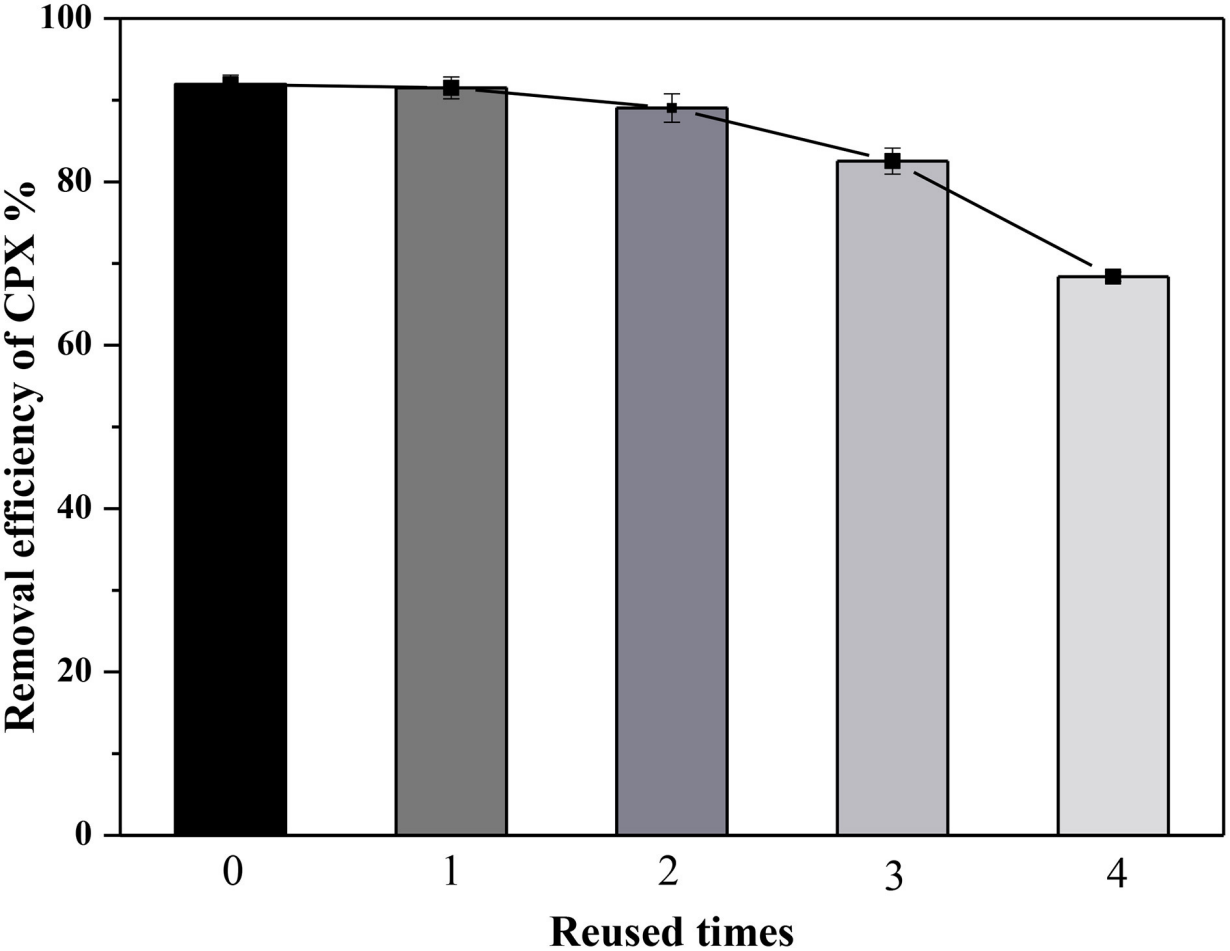
Regenerated use of Fe-MCM-41 adsorbent for removal of CPX (volume of CPX solution: 200 mL; rotating speed: 175 rpm/min; initial pH-value: 5.40; equilibrium time: 120 min).

It was important for an adsorbent with good reuse performance in practical application. According to the investigation of pH influence, the adsorption capacity of CPX on Fe-MCM-41 was negligible when the pH was above 10. It indicated that of CPX could desorbed from the absorbent surface using 0.1 M NaOH solution as the desorption agents possibly, then the adsorbent was filtered, washed and dried at 358 K. The regenerated adsorbent was tested by four cycles of adsorption/desorption process under the same conditions. Figure 11 presented the removal efficiency of CPX on the recycled Fe-MCM-41, exhibiting slightly decline with a loss of 23.6% after four cycles, which might due to the damage of adsorption sites. Therefore, Fe-MCM-41 could be recycled as an efficient absorbent for practical application.

## Conclusions

Fe-MCM-41s prepared from a hydrothermal process were used as adsorbents to adsorb the CPX in aqueous solutions. Experimental results described that Fe-MCM-41 with Si/Fe = 20 exhibited the best CPX removal efficiency. The XRD and TEM results confirmed that the hexagonal mesoporous structure maintained after iron doping. A combination of multiple effects including electrostatic interaction, surface complexation, hydrophobic effect and π-π interaction on the adsorption process were confirmed under different reaction conditions. The experimental kinetic data showed that the adsorption was a chemical-controlling and multi-step process, Fe-MCM-41 showed the better adsorption property. Strong acidic/alkaline environment was not conducive to the adsorption process. The adsorption performed a higher adsorption affinity at higher temperature was a spontaneous exothermic process. Moreover, due to the depression of Me-CPX complex, metal cations decreased CPX adsorption on Fe-MCM-41 surface. As the same order as the complexation stability constants of CPX, Cu impacted CPX adsorption most. Fe-MCM-41 exhibited stable performances for 4 cycles use without deterioration which could possibly applied to wastewater treatment including POPs.

## Author contributions

YW: Conducted all the experiments; YT: Supervised the project; LL: Revised the manuscript; PL and XL: Provided assistance in setting up the gears; WC and YX: Helped analysis the data.

### Conflict of interest statement

The authors declare that the research was conducted in the absence of any commercial or financial relationships that could be construed as a potential conflict of interest.
