# Supplementary material for: The Correlation of Adsorption Behavior between Ciprofloxacin Hydrochloride and the Active Sites of Fe-doped MCM-41
Source: Front Chem. 2018 Feb 7;6:17. doi: 10.3389/fchem.2018.00017 (PMC5808354; doi:10.3389/fchem.2018.00017)
Supplement: Supplementary file 1 [file DataSheet1.DOC]

Supplementary Material

# The correlation of adsorption behavior between ciprofloxacin hydrochloride and the active sites of Fe-doped MCM-41

**Ying Wu, Yiming Tang*, Laisheng Li, Peihong Liu, Xukai Li, Weirui Chen, Ying Xue**

*** Correspondence:** Yiming Tang: [y.tang@m.scnu.edu.cn](mailto:y.tang@m.scnu.edu.cn)

# Supplementary Figures and Tables

## Supplementary Figures


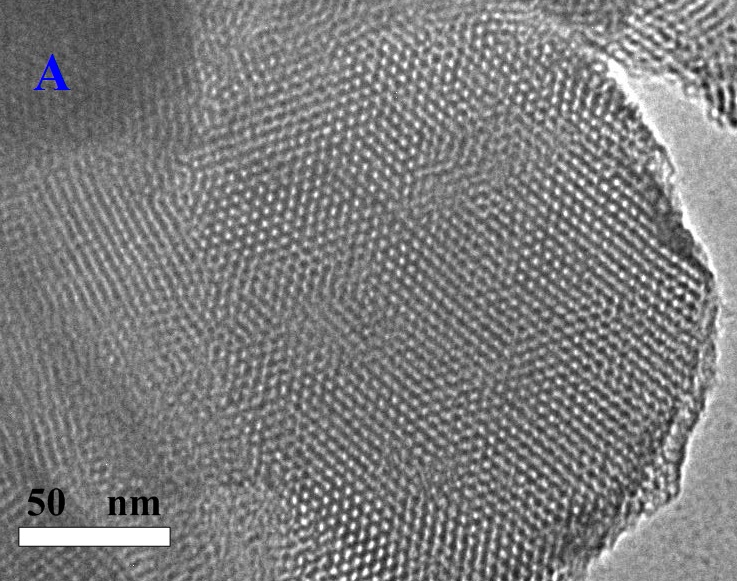

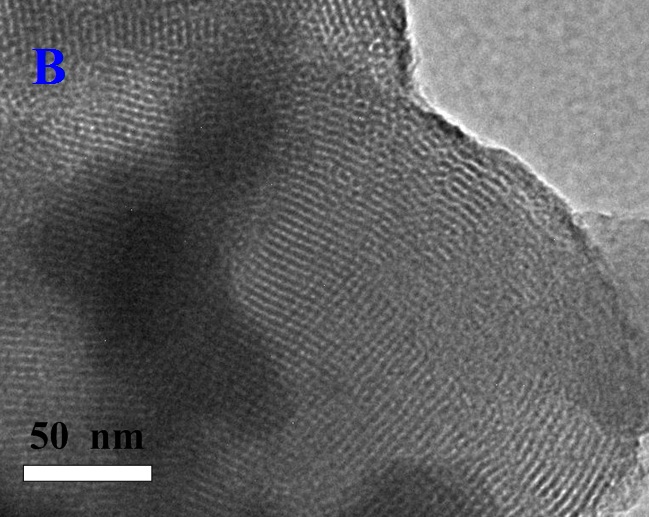


**Supplementary Figure S1.** TEM images of pure MCM-41 (A) and Fe-MCM-41(20) (B).

**Supplementary Figure S2.** FT-IR spectra of CPX, Fe-MCM-41 and CPX-adsorbed Fe-MCM-41over the range 4000-400 cm^-1^.

## Supplementary Tables

**Supplementary Table S1.** Structural properties of adsorbents.

| **Sample** | **S_BET_** **(m^2^ g^-1^)** | **Pore diameter (nm)** | **Pore Volume (cm^3^ g^-1^)** |
| --- | --- | --- | --- |
| MCM-41 | 1002.1 | 3.2 | 0.79 |
| Fe-MCM-41 (160) | 894.7 | 3.5 | 0.79 |
| Fe-MCM-41 (80) | 883.2 | 3.6 | 0.80 |
| Fe-MCM-41 (40) | 822.6 | 3.8 | 0.79 |
| Fe-MCM-41 (20) | 625.6 | 4.3 | 0.68 |
